# Research Progress on the Mechanism of Mitochondrial Autophagy in Cerebral Stroke

**DOI:** 10.3389/fnagi.2021.698601

**Published:** 2021-07-15

**Authors:** Li Lei, Shuaifeng Yang, Xiaoyang Lu, Yongfa Zhang, Tao Li

**Affiliations:** ^1^Department of Neurosurgery, The First People’s Hospital of Yunnan Province (Kunhua Hospital/The Affiliated Hospital of Kunming University of Science and Technology), Kunming, China; ^2^Translational Neurosurgery and Neurobiology, University Hospital Aachen, RWTH Aachen, Aachen, Germany

**Keywords:** mitochondrial autophagy, PINK1/Parkin, FUNDC1, NIX/BNIP3, cerebral stroke

## Abstract

Mitochondrial autophagy is an early defense and protection process that selectively clears dysfunctional or excessive mitochondria through a distinctive mechanism to maintain intracellular homeostasis. Mitochondrial dysfunction during cerebral stroke involves metabolic disbalance, oxidative stress, apoptosis, endoplasmic reticulum stress, and abnormal mitochondrial autophagy. This article reviews the research progress on the mechanism of mitochondrial autophagy in ischemic stroke to provide a theoretical basis for further research on mitochondrial autophagy and the treatment of ischemic stroke.

## Introduction

Stroke is a sudden disease with disturbance of cerebral blood circulation, which has surpassed ischemic heart disease, lung cancer, chronic obstructive pulmonary disease, and liver cancer to become the disease with the highest age-standardized mortality rate in China. According to the latest disease burden report in 2015, stroke has become not only the first cause of disease burden, but also the second leading cause of death and disability in the world (GBD 2015 DALYs and HALE Collaborators, 2016; GBD 2015 Mortality and Causes of Death Collaborators, 2016), and an important cause of premature death. Stroke is primarily divided into two categories: ischemic stroke (accounting for 60% of all strokes) and hemorrhagic stroke (Krishnamurthi et al., 2013). At present, studies have found a substantial link between mitochondrial dysfunction and the occurrence and development of ischemic stroke. Moreover, the abnormal autophagy is profoundly involved in mitochondrial dysfunction.

## Autophagy

Autophagy is a process defined by the degradation of abnormal proteins and damaged organelles by bilayer membranes such as exfoliated endoplasmic reticulum or Golgi complex under physiological or pathological circumstances and mediated by autophagy-related proteins. The bilayer structures fuse with lysosomes to form autophagy lysosomes, which degrades the contents of biological macromolecules and organelles, so as to maintain cell homeostasis by meeting the needs of cellular metabolism and organelle renewal (Parzych and Klionsky, 2014; Tagaya and Arasaki, 2017). Autophagy is essential for neuronal survival in the central nervous system. According to the mechanism and the mode of action, autophagy can be divided into three types: macroautophagy, microautophagy, and chaperone-mediated autophagy (CMA). Among them, macroautophagy is the most thoroughly studied. Mitochondrial autophagy, also called mitophagy, is a kind of macrophage autophagy, which selectively degrades damaged mitochondria through autophagy mechanism to sustain the stability of the intracellular environment. Mitochondrial autophagy plays an important role in regulating cellular homeostasis, proliferation, differentiation, genetics, aging, and cell death, as well as in controlling the quantity and quality of mitochondria (Glick et al., 2010).

## The Regulatory Mechanism of Mitochondrial Autophagy

### PTEN Induced Putative Kinase 1 (PINK1)/Parkin Signaling in Mitochondrial Autophagy

**Figure 1:**
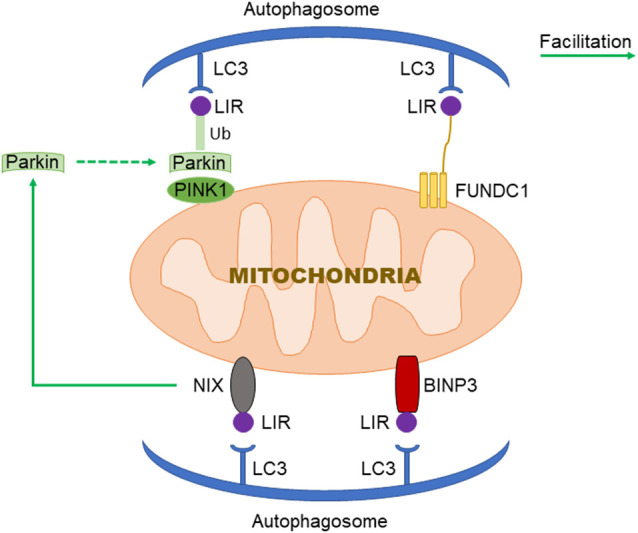
Three signaling pathways regulating mitochondrial autophagy. In PTEN Induced Putative Kinase 1 (PINK1)/Parkin pathway, PINK1 functions as the initiator which recognizes the disturbed outer membrane potential and activates Parkin and ubiquitin phosphorylation, and then the complex is combined by LC3 to autophagosome; FUN14 Domain-Containing 1 (FUNDC1) is a three transmembrane protein having the LC3 interaction region (LIR) motif on the N-terminal which can be recognized by LC3; both BNIP3 and NIX have LIR motif, additionally, NIX can facilitate the localization of Parkin to the damaged mitochondria.

PTEN Induced Putative Kinase 1 (PINK1)/Parkin-mediated autophagy is the most common type of mitochondrial autophagy in mammalian cells (He et al., 2019). PINK1 is a nuclear-encoded mitochondrial serine (Ser)/threonine kinase (Eiyama and Okamoto, 2015; Quinn et al., 2020), which is divided into N-terminal mitochondrial targeting signal (MTS), α-helical transmembrane domain (TM), serine/threonine kinase domain, and C-terminal mitochondrial outer membrane retention signal peptide sequence (Deas et al., 2011), it can mediate the ubiquitination of substrates, and can regulate protein degradation and signal transduction. Parkin is an E3 ubiquitin ligase encoded by the Park2 gene. Genetic studies have shown that PINK1 plays a role in the upstream of Parkin, and PINK1 and Parkin are in the same pathway to preserve mitochondrial functions (Poole et al., 2008; Whitworth and Pallanck, 2009; Tanaka, 2020). In healthy cells, PINK1 is transported to the mitochondrial inner membrane, where it is cut and degraded rapidly by the ubiquitin-protease system, when the intracellular mitochondrial membrane is damaged, and consequently, the transmembrane potential is disturbed, the transport of PINK1 into the mitochondria will be blocked and PINK1 will accumulate in the outer membrane of the mitochondria, which then mediates the activation of Parkin and ubiquitin phosphorylation (Poole et al., 2008; Jin and Youle, 2013; Durcan and Fon, 2015; Okatsu et al., 2015a; Yamano et al., 2016). PINK1 can simultaneously phosphorylate Ser65 in the N-terminal ubiquitin-like domain of Parkin and produce Ser65 phosphorylated Parkin (Kondapalli et al., 2012; Iguchi et al., 2013; Okatsu et al., 2015b; Yang et al., 2020), phosphorylated Ser65 activates its ubiquitin ligase activity, which is able to connect the polyubiquitin chain to the mitochondrial outer membrane protein (Kazlauskaite et al., 2014), the complex is recognized by LC3, binds to the autophagosome, and then fuses with the lysosome to form the autophagy lysosome that finally degrades the damaged mitochondria. Studies from the Boule laboratory have confirmed that PINK1/Parkin-regulated mitochondrial autophagy plays a key role in the clearance of damaged mitochondria (Lazarou et al., 2015). Mitochondria maintain metabolic balance through continuous fusion and division. Additionally, the fusion/division and the mitochondrial autophagy influence each other; malfunctioning mitochondria are effectively removed by mitochondrial autophagy, and only the mitochondria with normal membrane potential can enter the normal fusion-division cycle (Twig et al., 2008), PINK1 and Parkin are of importance in the process of mitochondrial fusion and division. Related studies have shown that PINK1 and Parkin can promote mitochondrial division and regulate mitochondrial movement by mediating Miro phosphorylation to isolate damaged mitochondria (Poole et al., 2008), maintaining energy balance, and avoiding oxidative stress (Zhuang et al., 2016; Figure 1).

### Mitochondrial Autophagy Regulated by FUN14 Domain-Containing 1 (FUNDC1)

FUN14 Domain-Containing 1 (FUNDC1) is a new type of mitochondrial membrane protein that mediates mitochondrial autophagy in mammalian cells, it mainly acts in controlling mitochondrial autophagy by regulating the phosphorylation level of FUNDC1 on the mitochondrial membrane. FUNDC1 is a protein located in the outer membrane of mitochondria, which contains three transmembrane domains, as well as the N-terminal domain exposed to the cytoplasm and the C-terminal domain inserted into the mitochondrial inner membrane. It has been found that FUNDC1 contains a typical LC3 interaction region (LIR) sequence consisting of amino acid residues at the N-terminal on the cytoplasmic side (Liu et al., 2014; Cai et al., 2021). Under physiological conditions, FUNDC1 inhibits mitochondrial autophagy due to the phosphorylation of Tyr18 in the LIR motif of sarcoma (Src) gene kinase, leading to the inability of FUNDC1 to recruit LC3 and to combine with it, which reduces the interaction between FUNDC1 and LC3 (Liu et al., 2012; Chen et al., 2016; Kuang et al., 2016). Studies have shown that mitochondrial autophagy receptor protein, FUNDC1, is involved in mitochondrial autophagy under the condition of hypoxia in mammalian cells (Wang et al., 2018), when Src kinase and CK2 (formerly known as casein kinase 2) are inactivated during hypoxia or mitochondrial uncoupling, Ser17 on FUNDC1 is dephosphorylated by unc-51-like kinase 1 (ULK1) and Ser13 is dephosphorylated by phosphoglycerol mutase family 5 (PGAM5; Liu et al., 2014; Chen et al., 2016, 2017; Kuang et al., 2016), enhancing FUNDC-LC3 interaction and mitochondrial autophagy (Imai et al., 2010; Liu et al., 2014; Lv et al., 2017). Thus, it can be seen that FUNDC1 phosphorylation or dephosphorylation functions as a specific mitochondrial autophagy receptor in the process of mitochondrial autophagy induced by hypoxia. In addition, FUNDC1 can regulate mitochondrial division-fusion and mitochondrial autophagy by interacting with mitochondrial mitotic protein, dynamin-related protein 1 (Drp1), and optic nerve dystrophin 1 or with Drp1 and calcitonin (Ding and Yin, 2012; Iguchi et al., 2013; Wu et al., 2016a).

### BNIP3/NIX Pathway in Mitochondrial Autophagy

BNIP3 (B Lymphoma-2 gene/adenovirus E1B interacting protein 3) and NIX (BNIP3L) are pro-apoptotic mitochondrial proteins located in the outer membrane of mitochondria (Kubli et al., 2007; Liu et al., 2019; Xu et al., 2019), they have 56% homology in amino acid sequence. Under certain conditions, the LIR sequence interacts with the recruited autophagy related protein, LC3, to form an autophagosome to clear the damaged mitochondria (Springer and Macleod, 2016; Šprung et al., 2018; Roperto et al., 2019). It has been reported that BNIP3/NIX mediates mitochondrial autophagy through upstream hypoxia-inducible 1 factor (HIF-1) regulation (Chourasia and Macleod, 2015); the competitive binding of BNIP3 and NIX to the anti-apoptotic protein, Bcl-2, will make Bcl-2-Beclin-1 complex dissociate and release Beclin-1, subsequently activating mitochondrial autophagy and reducing the apoptosis (Zhang et al., 2008). BNIP3 and NIX can promote the enhancement of mitochondrial autophagy by inhibiting the mammalian rapamycin target protein (mTOR) to activate protein Rheb. The upregulation of BNIP3 and NIX expression can be mediated by forkhead box O3 (FOXO3). Some studies have found that BNIP3 and NIX can not only induce cell death, but also increase their expression level under hypoxia, reducing reactive oxygen species (ROS) production and promoting cell survival, and can participate in the regulation of mitochondrial autophagy (de Vries and Przedborski, 2013). BNIP3 can increase the localization of mitochondrial kinesin-associated protein 1, thus promoting the phagocytosis of damaged mitochondria (Liu, 2015). Additionally, BNIP3 promotes the release of cytochrome C. Other studies have found that mitochondrial oxidative phosphorylation uncoupling agent carbonyl cyanide m-chlorophenyl hydrazone (CCCP) treatment on Hela cells can improve the interaction between NIX and LC3, indicating that NIX may also be involved in depolarization-induced mitochondrial autophagy (Novak et al., 2010). NIX mainly regulates mitochondrial autophagy at baseline under physiological conditions and mediates mitochondrial clearance during erythrocyte development by increasing the production of ROS (Novak and Dikic, 2011; Shi et al., 2014). Moreover, NIX can promote the localization of Parkin to the damaged mitochondria, increase the ubiquitination of mitochondrial membrane proteins by Parkin, and eventually induce mitochondrial autophagy through the interaction between p62 and LC3 (Geisler et al., 2010).

## Mitochondrial Autophagy and Stroke

**Figure 2:**
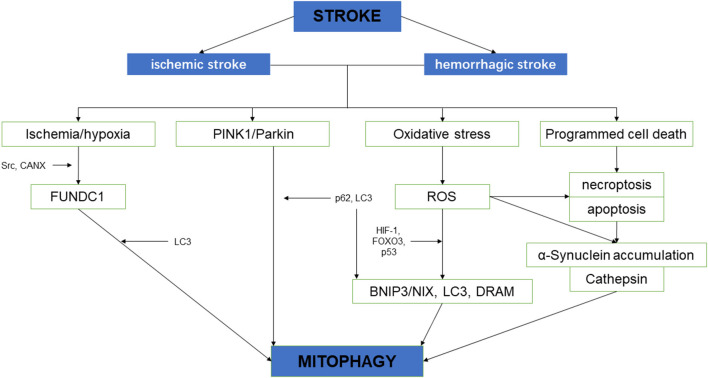
The schematic diagram depicting the relationship between cerebral stroke and mitochondrial autophagy.

Neurological impairment caused by mitochondrial dysfunction is significantly associated with stroke, the main mechanisms include increased adenosine triphosphate (ATP), increased ROS level, and oxidative stress overload. Under mild ischemia or tolerable hypoxic stress, mitochondrial autophagy can benefit cell homeostasis and promote cell survival. On the contrary, vulnerable brain cells in the ischemic core area of persistent ischemia or reperfusion begin to die, which will lead to excessive long–term autophagy hyperactivity, causing cell damage or death (Zhang X. et al., 2013). Therefore, in the course of a stroke, it may be a possible solution of protecting neurons and preventing cell death to regulate the quantity and quality of mitochondria through the process of mitochondrial autophagy (Wong and Cuervo, 2010; Figure 2).

### ROS and Stroke

Mitochondria are vital organelles that produce energy, regulate cell signals and apoptosis, and are also the major source of ROS (Murphy, 2009). When cerebral ischemia occurs, the decrease of blood flow leads to oxygen and glucose deficiency and changes in mitochondrial structure. Excitatory amino acid toxicity, hypoxia, calcium overload, and other factors cause mitochondrial oxidative phosphorylation disorder (Li et al., 2015; Khandelwal et al., 2016), thus the excessive ROS produced by damaged nerve cells cannot be eliminated by related enzymes. In turn, it results in the destruction of the redox balance of lipids and proteins and extensive cell damage, which further aggravates the mitochondrial damage. Increased level of ROS-enhanced oxidative stress response and excessive generation of ROS can activate hypoxia-inducible factor 1 (HIF-1) and FOXO3, these transcription factors then induce the transcription of BNIP3 and NIX, and LC3 and BNIP3, respectively (Li et al., 2015). HIF-1 is the main factor in the survival response of cells to hypoxia, which can induce the transcription of BNIP3 and NIX genes. Their protein products compete with Beclin-1 to bind Bcl-2 to release Beclin-1 and allow it to induce autophagy (Mahalingaiah and Singh, 2014). It has been found that the increase of intracellular ROS may trigger the FOXO3 signaling pathway, which activates the transcription of ubiquitin-proteasome pathway and autophagy-related genes (such as the genes encoding LC3 and BNIP3), thus inducing autophagy (Aucello et al., 2009). After 3 days of ischemia/reperfusion, superoxide dismutase (SOD) and glutathione peroxidase (GSH-Px) in brain tissue decreased significantly. With the intervention using Salvianolic acid, oxygen-free radicals were effectively scavenged in rats, and ischemia-reperfusion injury was significantly suppressed (Xu et al., 2017). Hypoxic preconditioning can activate the HIF-1/Beclin-1 signaling pathway to facilitate autophagy and protect against ischemic and hypoxic injury (Semenza, 2011; Lu et al., 2018). When cells were exposed to hypoxia, the transcriptional level of BNIP3/NIX was directly regulated by HIF-1α and activated (Tracy et al., 2007). Bellot confirmed in a variety of cell types that hypoxia promoted the inhibition of proline hydroxylase (PHD) activity through ROS produced by mitochondria (Bellot et al., 2009), suppressed the degradation of HIF-1α, and up-regulated the expression of autophagy genes such as Beclin-1 and Atg5 to promote mitochondrial autophagy (Jin and Youle, 2012). ROS not only induces mitochondrial autophagy through the HIF-1 signaling pathway (Dias et al., 2013), but also facilitates mitochondrial autophagy by activating the PINK1/Parkin pathway. In addition, ROS can induce autophagy by inhibiting the activity of protein kinase B (Akt) and mTOR in the PI3K/Akt/mTOR signaling pathway (Mellor et al., 2011; van der Vos et al., 2012; Zhang J. et al., 2013; Fiorini et al., 2015; Hambright et al., 2015; Duan et al., 2016).

### The PINK1/Parkin Signaling Pathway and Ischemic Stroke

The PINK1-Parkin pathway is one of the typical signaling pathways in mitochondrial autophagy. Under physiological conditions, PINK1 and Parkin form a strict mitochondrial regulation mechanism and keep a mutual balance, preventing injury of autophagy caused by excessive mitochondrial autophagy. During mitochondrial dysfunction caused by ischemic stroke (Lazarou et al., 2012; Wauer et al., 2015), the PINK1/Parkin pathway mediates the polyubiquitin of the functional or structural proteins in the damaged mitochondria (Riley et al., 2013; Trempe et al., 2013; Kumar et al., 2015; Okatsu et al., 2015b; Yamano et al., 2015). PINK1 can quickly sense the membrane potential abnormality of the damaged mitochondria, recruit Parkin to gather to the damaged ones, and induce mitochondrial autophagy (Lee et al., 2010). In recent years, there has been an increase in the research on this mechanism in stroke, which illustrates to some extent that the PINK1-Parkin pathway is involved in the process of brain injury in stroke. Safarpour’s studies on ischemic stroke using neurons found that knockout of PINK1 or Parkin gene could result in excitatory amino acid toxicity to the neurons, and it was confirmed that either PINK1 or Parkin gene deletion could aggravate neuronal damage in ischemic stroke. Wu et al. (2018) established the ischemic stroke cell model using rat hippocampal neurons *in vitro*. The results showed that PINK1/Parkin-mediated mitochondrial autophagy could play a neuroprotective role in rat hippocampal neurons. It was also revealed that H2 and rapamycin could increase cell survival, increase the expression of LC3-II, PINK1 and Parkin, reduce the loss of mitochondrial membrane potential, and inhibit the level of ROS and the rate of apoptosis. After autophagy inhibitor 3-methyladeine (3-MA) treatment, mitochondrial autophagy was inhibited, and the cell survival was significantly downscaled, accompanied by a significant increase in the level of ROS and the rate of apoptosis. It was found that in the pathophysiological process of ischemic stroke, PINK1/Parkin signaling pathway exerted a protective effect by mediating mitochondrial autophagy; it was also reported that the expression of LC3B and Beclin-1 was gradually up-regulated and reached the peak 24 h after cerebral ischemia-reperfusion, and the mitochondrial translocation of Parkin and the expression of PINK1 in mitochondrial outer membrane increased significantly at 24 h of reperfusion. When treated with a PINK1 inhibitor, the expression of LC3B and Beclin-1 is decreased, the mitochondrial translocation of Parkin is lessened, and the volume of cerebral infarction is increased significantly. In addition, laboratory studies have proven that in the course of ischemic brain injury, silencing the Parkin gene can interfere with mitochondrial autophagy induced by OGD (oxygen-glucose deprivation) reperfusion model, and further aggravate neuronal injury.

### Mitochondrial Autophagy and Cerebral Stroke Regulated by NIX/BNIP3

Under normal conditions, the expression of BNIP3/NIX is low in most organs, but under hypoxic-ischemic conditions, its transcriptional level is directly regulated by HIF-1α and activated (Tracy et al., 2007; Semenza, 2011; Guo, 2017; Yuan et al., 2017). Neuronal excitatory injury can induce mitochondrial autophagy during cerebral ischemia (Borsello et al., 2003; Shacka et al., 2007), which might occur *via* up-regulation of the expression of p53 and damage regulatory autophagy modulator DRAM(an autophagy regulator), and in turn, modulating autophagy by influencing Beclin-1 and LC3 (Rami et al., 2008; Wang et al., 2009; Poluzzi et al., 2014; Maejima et al., 2016). Mitochondrial autophagy can play a neuroprotective role by inhibiting apoptosis in the model of transient ischemia and hypoxia, in which the expression of Beclin-1 protein and the ratio of LC3-II/I is increased, while the expression of p62, TOM20, and HSP60 is decreased, and the progression of cerebral infarction is mild (Huang et al., 2016). In the model of long-term ischemia or permanent middle cerebral artery occlusion (MCAO), the detection of mitochondrial autophagy-related proteins, including Beclin-1, LC3, p62, TOM20, and HSP60 also shows that mitochondrial autophagy is inhibited and that the volume of cerebral infarction increases significantly. However, overactivated autophagy induces neuronal apoptosis and brain tissue injury (Li et al., 2015). Autophagy inhibitor 3-MA exerts the neuroprotective effect by inhibiting the up-regulation of LC3-II and cathepsin B induced by ischemia, preventing the programmed cell death of hippocampal CA1 neurons, and thus reducing the infarct volume and brain edema (Kubota et al., 2010). It is confirmed that the inhibition of Beclin-1 can reduce the cell death caused by MCAO (Xing et al., 2012). Recent research shows that necrostatin-1 treatment suppresses autophagic-associated proteins (LC3-II, Beclin-1) and maintains p62 at a normal level at 24 and 72 h after intracerebral hemorrhage (ICH) in a mouse model; the study also proves that the specific inhibitor, necrostatin-1, inhibits apoptosis and autophagy to exert the neuroprotective effects after ICH (Sekerdag et al., 2018). Zhang et al. (2011) reported that atorvastatin (ATV) weakens the neuroprotective effect of endoplasmic reticulum stress-related apoptosis through the reduction of autophagy in MCAO rats (Yuan et al., 2017), suggesting that compounds that inhibit autophagy may reduce the neuroprotective effect of angiotensin after cerebral ischemia (Adhami et al., 2006). Rapamycin, an inhibitor of mTOR, can activate autophagy after permanent ischemia and ischemia-reperfusion, increase the phosphorylation of Akt and cAMP response element binding protein (CREB), reduce infarction volume, improve stroke prognosis, and participate in protection against cerebral ischemic injury (Sheng et al., 2010). Carloni et al. (2010) used Rapamycin in the neonatal hypoxic-ischemic brain damage model and found that the expression of Beclin-1 was significantly up-regulated and the death of hippocampal and cortical neurons decreased significantly. The study speculated that drugs that appropriately up-regulate autophagy may have a certain neuroprotective effect in patients with large area cerebral infarction or transient ischemic attack (TIA; Carloni et al., 2008). Zheng et al. (2009) applied the ribonucleic acid (RNA) interference technique to the rat model of cerebral ischemia to down-regulate the expression of Beclin-1 in the ischemic brain and inhibit mitochondrial autophagy and noted that it can alleviate cerebral ischemic injury, inhibit neuronal apoptosis in cortex and striatum, and improve the symptoms of neurological impairment. Recent studies have shown that the re-expression of BNIP3L in mouse cortex and striatum transfected with recombinant adeno-associated virus can reverse the loss of BNIP3L after ischemia and promote mitochondrial autophagy. Additionally, this study found that only high expression of wild-type BNIP3L could reverse mitochondrial autophagy and reduce ischemic cerebral infarction volume after cerebral ischemia, whereas high expression monomer mutant BNIP3L could not have such effect. These results suggest that BNIP3L dimer formation is necessary to induce the mitochondrial autophagy to exert a neuroprotective effect, and the decrease of BNIP3L dimer is a key factor for the loss of mitochondrial autophagy in ischemic brain tissue. In ischemia and reperfusion, when the Parkin gene is knockout *in vivo* and *in vitro*, BNIP3L is still able to promote mitochondrial autophagy, and the mitochondrial autophagy-related proteins such as Parkin, FUNDC1, Bcl2-L-13, Prohibitin2, and BNIP3L/NIX can be detected. It was suggested that only the protein level of BNIP3L/NIX decreased with the prolonged duration of ischemia time. Recently, a series of studies have reported that tumor suppressor gene p53 and DRAM have an important relationship with the occurrence of autophagy during cerebral ischemia. Nerve excitatory injury can up-regulate the expression of p53 and DRAM, and then induce autophagy by regulating Beclin-1 and LC3 (Wang et al., 2009; Zhang et al., 2009; Sano et al., 2020).

### The Ischemic Stroke Regulated by FUNDC1

FUNDC1 is a membrane protein located in mitochondria. FUNDC1 binds to LC3 through its LIR domain, causing an autophagic bilayer membrane to envelop mitochondria, inducing mitochondrial autophagy. The resulting mitochondrial autophagy inhibits apoptosis and protects neurons. FUNDC1 is a bridge between tissue-type plasminogen activator (tPA)-regulated apoptosis and mitosis in ischemia-reperfusion injury (IR) damage (Cai et al., 2021; Li et al., 2021). The tPA plays an important role in the treatment of acute cerebral ischemic injury, it has the thrombolytic effect in blood vessels and is also an important neuroprotective agent in the ultra-early ischemic stroke (Jeanneret and Yepes, 2017; Zhou et al., 2018; Yepes, 2019). FUNDC1 is associated with mitosis and is widely involved in the course of IR. The tPA exerts neuroprotective effects by increasing the phosphorylation of AMPK and the expression of FUNDC1, thereby inhibiting apoptosis and improving mitochondrial function. Knocking out the tPA gene significantly aggravated brain injury and increased neuronal apoptosis and mitochondrial damage (Echeverry et al., 2010; Wu et al., 2012; An et al., 2014). Mitochondrial oxidative stress induced by blood re-flow after ischemia can lead to mitochondrial dysfunction and promote the release of cytochrome and Bcl family members (Cai et al., 2021). Recent studies have uncovered that FUNDC1 aggregates on the mitochondrial membrane by mediating the interaction between FUNDC1 and CANX protein on ER under anoxic conditions. Under hypoxic stress, the process of mitochondrial autophagy is activated. The separation of FUNDC1 from CANX, and the recruitment of DNM1L/Drp1 then lead to mitochondrial division (Wu et al., 2016b).

## Summary and Outlook

Mitochondria are the key target of cerebral stroke research. As a fundamental organelle, mitochondria play a pivotal role in the fate of neurons suffering an ischemic or hemorrhagic attack, causing neuronal death through a variety of biochemical and molecular processes. In recent years, there are numerous studies with respect to the correlation of cerebral stroke and mitochondrial autophagy, and also some preclinical studies aimed at interfering with autophagy to treat central nervous system diseases (Papadakis et al., 2013; Salminen et al., 2013). However, there is still a shortage of research on mitochondrial autophagy and hemorrhagic stroke, and it is an undeniable fact that the current results show some contradictions. Although the exact role of mitochondrial autophagy in stroke is controversial, it may also become a new hot spot in basic research and potential clinical application. It has been confirmed that drugs such as metformin, resveratrol, ginkgetin, and ezetimibe can activate autophagy to exert an anti-ischemic effect. For example, resveratrol protects the brain from NLRP3 injury by inhibiting the activation of NLRP3 inflammatory bodies through Sirt1-dependent autophagy; ginkgetin can alleviate cerebral ischemia/reperfusion induced autophagy and apoptosis by inhibiting the NF-κB/p53 signaling pathway (He et al., 2017; Yu et al., 2018; Pan et al., 2019). However, due to a lack of studies on drug side effects in experimental animals and the results of clinical trials, the conclusion that if autophagy modulating drugs mediate cytoprotection or cytotoxicity in cerebral stroke remains unavailable. We believe that the in-depth study of the molecular biological mechanism of mitochondrial autophagy will provide new perspectives, treatment strategies, and potential therapeutic targets for stroke and other diseases.

## Author Contributions

LL, SY, and XL presented the idea and wrote the manuscript. YZ and TL revised the article and are corresponding authors. All authors contributed to the article and approved the submitted version.

## Conflict of Interest

The authors declare that the research was conducted in the absence of any commercial or financial relationships that could be construed as a potential conflict of interest.
